# A circovirus-like genomic sequence with unique architecture and phylogenetic relatedness to human-linked viral lineages

**DOI:** 10.3389/fcimb.2025.1698076

**Published:** 2025-12-11

**Authors:** Liyan Dai, Lichen Mao, Wenhui Shi, Alfred Ndjekadom, Xiaochun Wang, Yuwei Liu, Shixing Yang, Likai Ji, Jianqiang Wang, Jin Ma, Wen Zhang, Quan Shen

**Affiliations:** 1 Department of Laboratory Medicine, School of Medicine, Jiangsu University, Zhenjiang, China; 2 Intensive Care Unit, Jintan District Hospital of Traditional Chinese Medicine, Changzhou, China; 3 Department of Laboratory Medicine, The Affiliated People’s Hospital, Jiangsu University, Zhenjiang, China

**Keywords:** circovirus, genomic structure, swine, cross-species transmission, viral metagenomics

## Abstract

Circoviruses are small viruses in the *Circoviridae* family that associate with a wide spectrum of disease from asymptomatic to lethal in different animal. In the current study, a novel swine-origin circovirus with unique genomic architecture was determined from both oropharyngeal swabs and fecal samples collected from febrile pigs in Jiangsu Province, China. The complete genome sequence of this novel porcine circovirus strain (named as PCV-CH-ZJ01) was determined to be 2544 nt in length, containing four major open reading frames: Rep1, 498–833 nt, 112 aa; Rep2, 1055–1546 nt, 164 aa; Cap1, 1756–2055 nt, 100 aa; Cap2, 2089–2529 nt, 147 aa. Comparative sequence analysis revealed differential conservation patterns across viral components and shared low amino acid similarity with other strains on the whole. Phylogenetic trees based the whole genome and amino acid of two Reps revealed that PCV-CH-ZJ01 belonged to circovirus and clustered with human and rhesus macaque circovirus strains, which raised the concerns of potential cross-species transmission between swine and human.

## 1 Introduction

Circoviruses, belonging to the family *Circoviridae*, are small, non-enveloped viruses characterized by the smallest known viral genomes among animal viruses, with circular single-stranded DNA genomes (approximately 2000 nucleotides [nt]) (Tischer et al., 1982; Hui et al., 2021; Wang et al., 2023; Gao et al., 2024). Generally, the viral genome exhibits an ambisense organization, harboring two large (>600 nucleotide) open reading frames (ORFs) which encode the replication-associated protein (Rep) and capsid protein (Cap) on complementary strands of a dsDNA replication intermediate formed during viral replication (Feher et al., 2023). Based on the phylogenetic analysis of Rep and Cap, members of the family are classified in one of two genera, Circovirus and Cyclovirus, according to the International Committee on Taxonomy of Viruses (ICTV) (Breitbart et al., 2017; Rosario et al., 2017).

Circoviruses exhibit extensive evolutionary adaptability, infecting diverse vertebrate hosts spanning animals (swine, cervids, bovines, canines, ursids, felids, mustelids), aquatic, and reptilian species, as well as human beings (Lorincz et al., 2011; Lorincz et al., 2012; Li et al., 2013; Bexton et al., 2015; Chiappetta et al., 2017; Nishizawa et al., 2018; Chang et al., 2020; Fisher et al., 2020; Zhang et al., 2020; Hui et al., 2021; Li et al., 2021). While infections manifest across a clinical spectrum from asymptomatic to lethal, porcine circoviruses (PCVs) represent the most consequential veterinary pathogens within this family. PCV2 and PCV3 specifically induce systemic inflammation, immunosuppression, and reproductive disorders in global swine populations, with PCV2-associated diseases alone causing huge economic losses in the pork industry. Recent metagenomic analyses have revealed some key discoveries: unprecedented viral diversity in non-porcine hosts and environmental reservoirs, and the detection of PCV in human, as well as phylogenetic evidence of cross-species transmission between avian and mammalian lineages. These findings raise public health concerns about zoonotic transmission of PCV to human.

Here we report the discovery and characterization of a novel circovirus identified through viral metagenomic analysis of porcine throat swabs and fecal samples. This emerging strain demonstrates a unique genomic architecture while maintaining significant genetic homology with known human circoviruses, and exhibits an alarmingly high prevalence rate in China swine populations.

## 2 Materials and methods

### 2.1 Sample collection

Viral screening was conducted through metagenomic analysis of 39 clinical specimens including 21 oropharyngeal swabs and 18 fecal samples collected from febrile pigs in Jiangsu Province, China during August 2022. To investigate the prevalence of the novel circovirus determined in this study in healthy pig herds, 326 fecal samples were collected in December 2023. Specimens were homogenized in phosphate-buffered saline (PBS; 1:10 w/v) and vortexed for 10 minutes. After a centrifugation at 15,000 × g for 15 minutes, the supernatants were transferred to new 1.5 ml tubes. The throat swab and fecal samples were combined into samples pools, respectively, and totally two sequencing samples pools were generated.

### 2.2 Viral metagenomic analysis

Viral metagenomic next-generation sequencing (NGS) was conducted as described before (Shen et al., 2022; Lu et al., 2025). Briefly, the procedure began by filtering 500 µL of each sample’s supernatant through a 0.45 µm membrane filter (Millipore, USA) to effectively remove large contaminants including eukaryotic cells and bacteria. Subsequently, the filtrates were digested with DNase I/RNase A (37°C, 1 h) to reduce the concentration of non-capsid protected nucleic acids. Following enzymatic treatment, viral nucleic acids were extracted using the QiaAmp Mini Viral RNA Extraction Kit (Qiagen, Germany) according to the manufacturer’s instruction. During the cDNA synthesis process, first-strand cDNA was generated using the SuperScript III First-Strand Synthesis System (Invitrogen, USA), and the second strand was synthesized using Klenow fragment DNA polymerase (TaKaRa, China), respectively. Thus, two independent libraries were constructed using the XT DNA Sample Preparation Kit and sequenced on the Illumina HiSeq platform with a dual-barcoding strategy.

### 2.3 Bioinformatics analysis

For bioinformatics analysis, the raw data produced by the HiSeq platform were processed according to the standard procedure which included debarcoding, trimming and assembling (Deng et al., 2015). Firstly, the raw 250 bp length of paired-end reads were debarcoded by vendor software from Illumina company. Reads were considered as duplicates if bases 5 to 55 were identical among them and one copy of duplicates was kept at random. Low quality tails were trimmed using Phred with the score of 10, and the clonal reads were removed. Adaptors were then removed using VecScreen with the default parameters, which is a system from NCBI for quickly identifying segments of a nucleic acid sequence that are of vector origin. The filtered clean reads were *De Novo* assembled by SOAPdenovo2 with a k-mer size of 63 and default parameters. The assembled contigs were aligned with a customized in-house viral proteome database using BLASTx with an E-value cutoff of 1×10^-5^.

### 2.4 PCR screening of novel circovirus strain

To determine the prevalence of the novel circovirus strain, a pair of PCR primers (Pigcir-F: 5’-GTGAAGAAAGGTAGTTTACAAGA-3’; Pigcir-R: 5’-ACTTTTACCGCATCCAGCGA-3’) were designed based on the conserved region of the circovirus genome, targeting a 491-bp amplicon. Viral DNA was extracted from plasma isolated from blood samples using the QIAamp Viral DNA Mini Kit (Qiagen, USA) following the manufacturer’s instructions. The amplification conditions were optimized as follows: initial denaturation at 94°C for 3 min, followed by 40 cycles of denaturation at 94°C for 30 sec, annealing at 55°C for 30 sec, and extension at 72°C for 90 sec.

### 2.5 Genome structure and phylogenetic analysis

The genome structure of this novel circovirus including potential open reading frames (ORFs) was identified using Geneious Prime and the NCBI ORFfinder tool. For phylogenetic analysis, the representative reference sequences based on ICTV were obtained from GenBank and multiple sequence alignments were carried out by MUSCLE with default parameters (Edgar, 2004; Breitbart et al., 2017). Phylogenetic trees based on complete genome, amino acid of Rep, and amino acid of Cap were subsequently constructed, repetitively, using the Maximum-likelihood (ML) method in MEGA 11, with 1000 bootstrap replicates.

## 3 Results

### 3.1 Viral metagenomic overview

The two libraries generated 90,350 and 1,776,682 clean sequence reads from oropharyngeal swab and fecal samples, respectively. Their average GC contents (GC%) were 54.0% and 45.0%, respectively. Following *de novo* assembly, 11,652 and 105,677 contigs were obtained for the oropharyngeal swab and fecal sample libraries. The contig N50 values were 319 for the oropharyngeal swab library and 407 for the fecal sample library.

### 3.2 Genome structure and comparative sequence analysis

**Figure 1:**
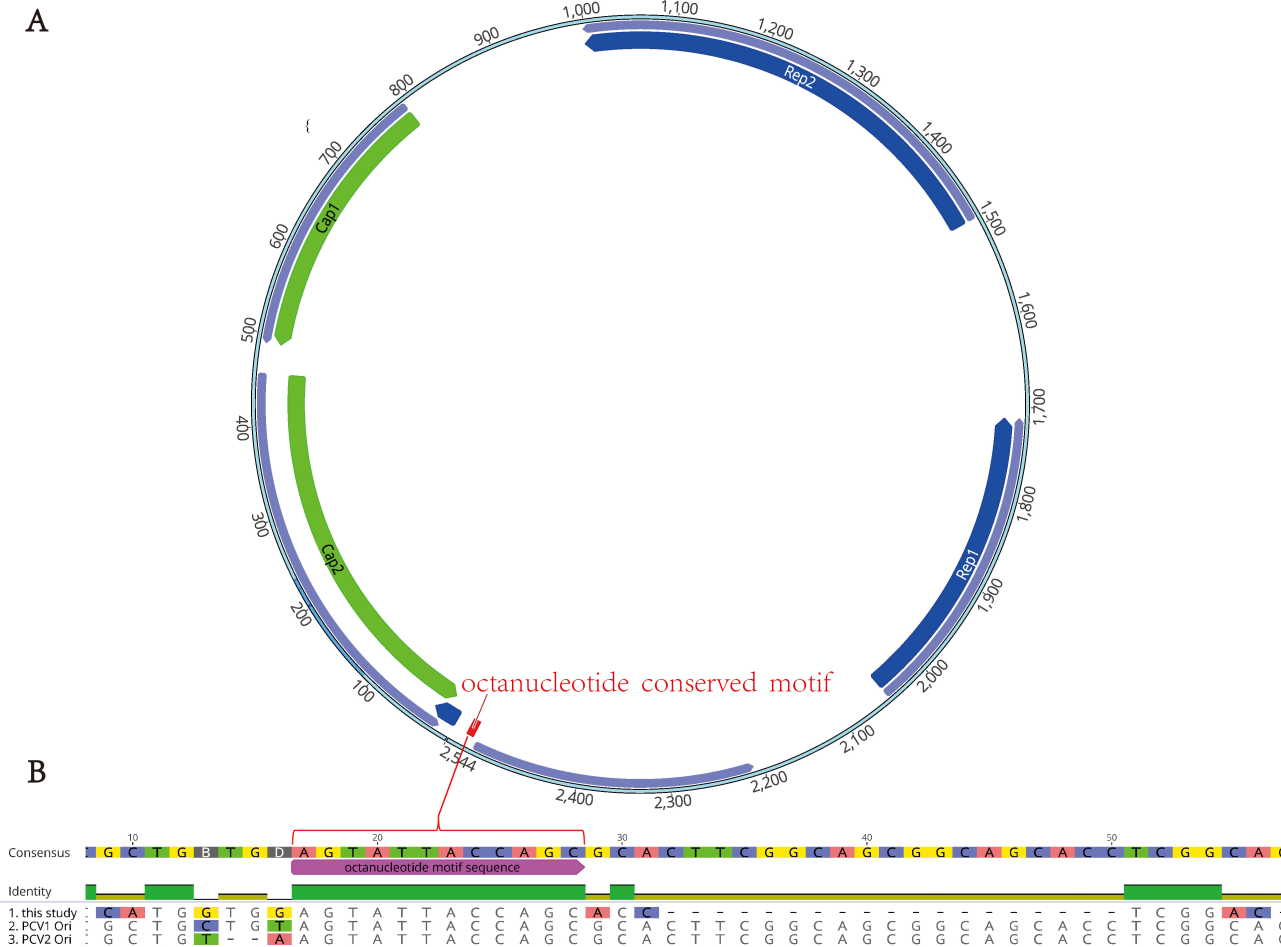
The unique genome structure of PCV-CH-ZJ01. **(A)** the four ORFs of PCV-CH-ZJ01, Rep1: 498–833 nucleotides, 112 amino acids; Rep2: 1055–1546 nucleotides, 164 amino acids; Cap1: 1756–2055 nucleotides, 100 amino acids; Cap2: 2089–2529 nucleotides, 147 amino acids. **B** an octanucleotide motif sequence of the Ori.

The complete genome sequence of porcine circovirus strain named as PCV-CH-ZJ01 was determined to be 2,544 nucleotides in length, containing four major open reading frames (**Figure 1**). These open reading frames encode two replicase proteins (Rep1: 498–833 nucleotides, 112 amino acids; Rep2: 1055–1546 nucleotides, 164 amino acids) and two capsid proteins (Cap1: 1756–2055 nucleotides, 100 amino acids; Cap2: 2089–2529 nucleotides, 147 amino acids). In alignment with characterized porcine circovirus strains encompassing PCV1 (GenBank accession no. AY094619) and PCV2 (GenBank accession no. AY184287), a conserved octanucleotide motif (AGTATTAC) demonstrating replication origin (ori) functionality was characterized within the genomic organization of PCV-CH-ZJ01 (**Figure 1B**) (Cheung, 2004).

Comparative sequence analysis revealed differential conservation patterns across viral components. At the nucleotide level, no significant homology was detected for Rep1 through BLASTn screening of the GenBank database. In contrast, Rep2 demonstrated 95.0% nucleotide identity with a human circovirus strain previously identified in Amerindian fecal specimens (MG571893). Similarly, Cap1 showed no database matches, while Cap2 exhibited limited genetic relatedness with 73.7% nucleotide identity to the aforementioned Amerindian-derived circovirus reference. Protein sequence comparisons showed distinct evolutionary relationships. The Rep1 protein displayed 78.5% amino acid identity to the replication-associated protein of a U.S.-origin rhesus macaque gut circovirus (KU043439), whereas Rep2 was found to share 95.7% identity with a primate-derived circovirus Rep protein from Uganda (USL90207). Notably, Cap1 failed to produce significant BLASTp matches due to low sequence conservation, and Cap2 was further characterized by limited homology (37.5% identity) to the capsid protein of the same rhesus macaque-associated strain (KU043439).

### 3.3 Phylogenetic analysis and molecular prevalence of the novel porcine circovirus

**Figure 2:**
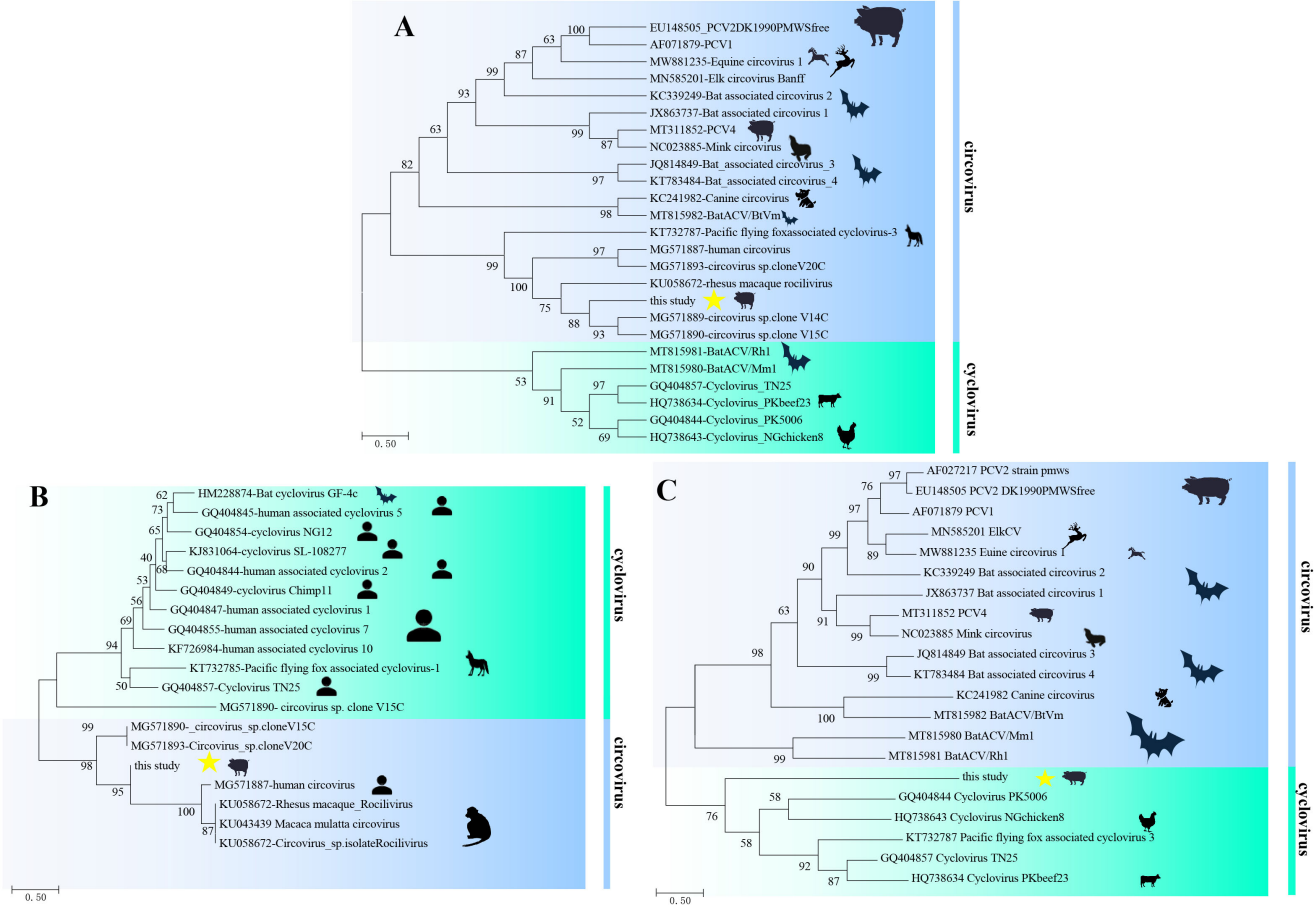
Phylogenetic trees of PCV-CH-ZJ01. These trees were constructed based on the complete genome nucleotide sequences **(A)**, as well as the amino acid sequences of Rep **(B)** and Cap **(C)**, using the maximum likelihood method with 1,000 bootstrap replicates.

Phylogenetic trees based on the whole genome and amino acid of two Reps (aa of Rep1+Rep2) revealed that PCV-CH-ZJ01 belonged to circovirus and clustered with human and rhesus macaque rhadinovirus strains (**Figures 2A, B**). In contrast, the phylogenetic tree based on the amino acid of the Caps protein unexpectedly grouped this novel PCV with cycloviruses (**Figure 2C**).

Result of PCR screening showed that the positive rate of this novel PCV in the fecal samples was 86.2% (281/326). According to the ICTV guidelines, circoviruses classified within the same species must exhibit nucleotide similarity of the complete genome exceeding 80% and a capsid protein amino acid similarity of over 70% (Zhang et al., 2020). Based on the aforementioned data, it becomes even more evident that the virus we have identified represents a totally novel PCV.

## 4 Discussion

The novel porcine circovirus was characterized by a unique bipartite genomic architecture, in which dual replication-associated proteins (Rep1/Rep2) and divergent capsid proteins (Cap1/Cap2) were identified. This structural divergence was evidenced by non-collinear gene arrangements compared to canonical circoviruses, along with the complete absence of homologous sequences for Cap1 in public databases. Phylogenetic clustering was observed to differ markedly from established circovirus lineages, with distinct branching patterns being supported across whole-genome, Rep-, and Cap-based trees. The relatively high bootstrap values of the clades comprising the PCV-CH-ZJ01 strain and human or non-human primate viruses indicate that the phylogenetic analysis was credible. Notably, Rep1 was found to share 79.35% amino acid identity with a circovirus isolated from rhesus macaques, while Rep2 exhibited 98% identity to a mammalian circovirus strain (GenBank USL90207.1). These primate-anchored sequence homologies were interpreted as molecular evidence of potential cross-species transmission between swine and primates, probably facilitated by ancestral recombination events or host-jumping processes.

High molecular prevalence of the virus was detected in healthy swine populations, with a 72.3% fecal shedding rate being recorded, which suggested that PCV-CH-ZJ01 may not be a highly pathogenic strain. The genetic novelty of Caps was hypothesized to alter receptor-binding specificity, and contribute to immune evasion. Nevertheless, the conserved mammalian motifs in Rep2 and primate-linked signatures in Rep1 were considered to warrant further investigation into zoonotic spillover risks, particularly given precedents of circovirus potential cross-species transmission. The samples used for library construction in this study were collected from febrile pigs on commercial farms; nevertheless, the samples employed to investigate the prevalence of PCV-CH-ZJ01 were obtained from pig herds without obvious clinical signs. Therefore, based on the available data, it is challenging to determine whether PCV-CH-ZJ01 induces significant disease or what its potential clinical manifestations might be. In our subsequent studies, we plan to isolate this strain and conduct animal experiments to assess its pathogenicity.

## Data Availability

The datasets presented in this study can be found in online repositories. The names of the repository/repositories and accession number(s) can be found in the article/supplementary material.
